# Early phosphoproteomic changes for adverse outcome pathway development in the fathead minnow (*Pimephales promelas*) brain

**DOI:** 10.1038/s41598-018-28395-w

**Published:** 2018-07-05

**Authors:** L. C. Smith, C. M. Lavelle, C. Silva-Sanchez, N. D. Denslow, T. Sabo-Attwood

**Affiliations:** 1 Department of Physiological Sciences, University of Florida, 1333 Center Dr., Gainesville, FL 32603 USA; 2 Department of Environmental and Global Health, University of Florida, 1225 Center Dr., Rm 4160, Gainesville, FL 32610 USA; 3 Center for Environmental and Human Toxicology, University of Florida, 2187 Mowry Rd, Gainesville, FL 32611 USA; 4 Interdisciplinary Center for Biotechnology Research, University of Florida, 2033 Mowry Rd, Gainesville, FL 32601 USA

## Abstract

Adverse outcome pathways (AOPs) are conceptual frameworks that organize and link contaminant-induced mechanistic molecular changes to adverse biological responses at the individual and population level. AOPs leverage molecular and high content mechanistic information for regulatory decision-making, but most current AOPs for hormonally active agents (HAAs) focus on nuclear receptor-mediated effects only despite the overwhelming evidence that HAAs also activate membrane receptors. Activation of membrane receptors triggers non-genomic signaling cascades often transduced by protein phosphorylation leading to phenotypic changes. We utilized label-free LC-MS/MS to identify proteins differentially phosphorylated in the brain of fathead minnows (*Pimephales promelas*) aqueously exposed for 30 minutes to two HAAs, 17α-ethinylestradiol (EE2), a strong estrogenic substance, and levonorgestrel (LNG), a progestin, both components of the birth control pill. EE2 promoted differential phosphorylation of proteins involved in neuronal processes such as nervous system development, synaptic transmission, and neuroprotection, while LNG induced differential phosphorylation of proteins involved in axon cargo transport and calcium ion homeostasis. EE2 and LNG caused similar enrichment of synaptic plasticity and neurogenesis. This study is the first to identify molecular changes *in vivo* in fish after short-term exposure and highlights transduction of rapid signaling mechanisms as targets of HAAs, in addition to nuclear receptor-mediated pathways.

## Introduction

The burden of toxicity assessments for the large number of chemicals on the market or coming to market has inspired new approaches to chemical prioritization, testing and regulation, globally. Adverse outcome pathways (AOP) are conceptual frameworks that organize and link contaminant-induced molecular changes to adverse biological responses at the individual and population levels. AOPs are widely accepted as powerful tools by regulatory agencies (e.g. Environmental Protection Agency and Organization for Economic Cooperation and Development) to improve regulatory decision-making through the integration of mechanistic data. Such developments allow for greater speed and accuracy of chemical testing while using fewer resources and experimental animals^1^. There are several events that comprise an AOP that include the initial interaction of a chemical with a biological target known as the molecular initiating event (MIE), followed by a series of sequential key events at the cellular, organismal and population levels that are indispensable and necessary for an adverse outcome. For example, activation of nuclear estrogen receptor alpha (ESR1) by xenoestrogens in fish is considered an MIE and such activation can initiate a series of key events including changes in expression of estrogen-responsive genes and alterations in circulating plasma sex steroids and vitellogenin. These biological responses have been further linked to reduced fecundity in females, altered gamete ratios in males, and reproductive behavioral deficits in both sexes that can ultimately lead to adverse population level effects^1^.

The development of AOPs is typically performed through a complement of bottom-up, top-down, and middle-out approaches. In bottom-up approaches, a chemical’s effect on a single MIE and a few genes, proteins, or biochemical reactions is studied at one time thus requiring prior knowledge of a chemical’s biological activity^2,3^. Top-down approaches start with a known adverse outcome and then delve deeper into lower levels of biological organization to identify the MIE and key events (KE)^2^. Middle-out approaches start with a phenotype or KE at the organism level that is not directly connected to an MIE or an AO but is subsequently connected by identifying the mechanisms underlying the change in the KE and linking that to a causal change leading to an AO^2,3^. Middle-out approaches thus enable discovery of new and essential components without prior knowledge^3^. Genomics, proteomics, and metabolomics technologies are particularly suited for middle-out approaches as a means to hypothesize, *a priori*, AOPs for toxicological processes of interest^1^, to ‘reverse engineer’ adverse effects observed in ecological targets exposed to contaminants with unknown mechanisms^4^, and to identify sub-lethal effects without a phenotypic anchor to parse adaptive pathways from toxicity pathways^5^.

Despite the increased utility of genomics, metabolomics, and proteomics in AOP development^6^, consideration of post-translational modifications (PTMs) on a global scale has been limited. Protein phosphorylation is a key PTM with 30–50% of proteins being phosphorylated in their lifetime^7^, and is typically induced by activation of membrane receptors that propagate rapid, non-genomic signaling cascades^8^. It is known that rapid signaling mediated by kinases in the brain is essential for gametogenesis in all vertebrate species^9^. For example, gonadotropin releasing hormone binds to the gonadotropin releasing hormone receptor in the pituitary, which triggers a string of sequential kinases including protein kinase C (PKC) and mitogen activated protein kinase/ extracellular signal-regulated kinase (MEK/ERK) by phosphorylation cascades. Activated MAPKs in turn phosphorylate cytosolic and nuclear proteins which initiate the transcription of genes including follicle stimulating hormone (FSH) and luteinizing hormone (LH) subunits and the release of the corresponding peptides into the blood where they can subsequently activate receptors in the gonad^9,10^.

Global, mass spectrometric-based phosphoproteomic methods have emerged as powerful tools for the non-targeted identification of phosphorylated proteins that can be used to identify targets of rapid, non-genomic signaling pathways. Although used modestly to assess the effects of environmental contaminants in human systems^11–13^ and in two studies focused on zebrafish development^14,15^, these methods have not translated to ecotoxicological assessment of contaminant exposure in non-mammalian species as a means to identify modes of action in target tissues or MIEs and key events in AOPs. Further, probing the phosphoproteome can provide additional mechanistic information that may be used in read-across analyses of chemicals with similar (or different) actions in the brain.

Here we characterize the brain phosphoproteome of male fathead minnows (FHM, *Pimephales promelas*) exposed through a water route separately to two constituents of the birth control pill, the synthetic estrogen, 17α-ethinylestradiol (EE2), or the synthetic progestin, levonorgestrel (LNG). We chose to investigate how these exposures promote rapid signaling in the brain as there is evidence to support roles for estrogen and progesterone in neurodevelopment in fish (reviewed by Pellegrini *et al*.^16^). We employed a label-free, semi-quantitative analysis of enriched phosphoproteins isolated from whole brains after 30 minutes of exposure to each compound. The short exposure period was used to identify rapid signaling networks in contrast to studies that have exposed FHM to these chemicals typically in the timeframe of 48 hours to 21 days and that target nuclear receptor-driven effects^17,18^. Results of this work represent the first evidence for rapid induction of molecular level changes by exposure to hormonally active chemicals in aquatic species, revealing that both EE2 and LNG cause rapid but differential changes in phosphorylation states of proteins involved in critical neuronal processes in the brain, thereby providing *a priori* knowledge for middle-out development of AOPs and to direct future studies investigating adverse phenotypic responses.

## Results

### Method Performance Analysis and Phosphoproteomic Profiling

**Figure 1:**
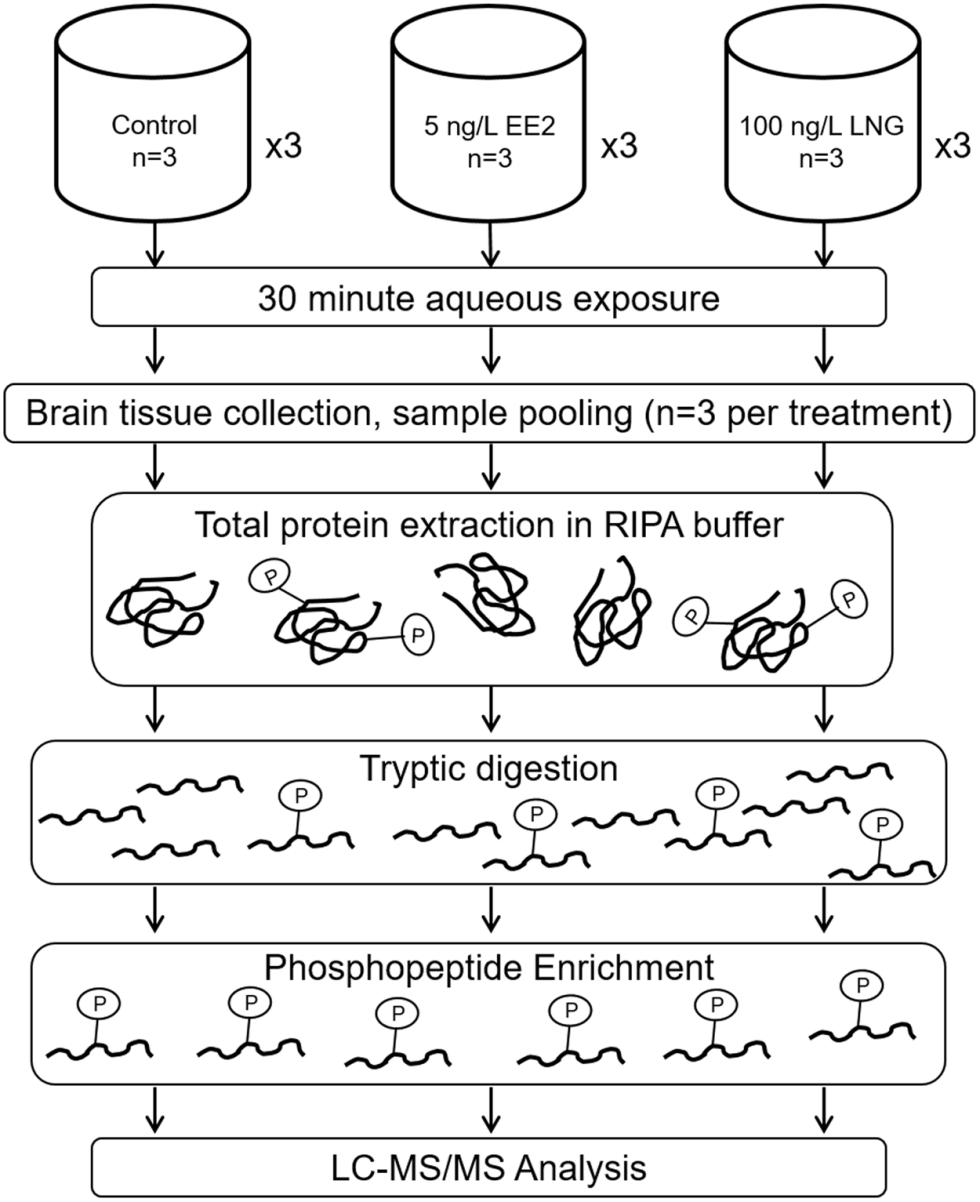
General phosphoproteomic workflow. FHMs were aqueously exposed to either vehicle control, 5 ng/L EE2, or 100 ng/L LNG for 30 minutes and the nine FHMs in each group were randomly assigned to one of three pools yielding n = 3 samples per exposure group. Purified total protein extracts were digested with trypsin and phosphopeptides were enriched using TiO_2_ columns and analyzed by LC-MS/MS.

**Figure 2:**
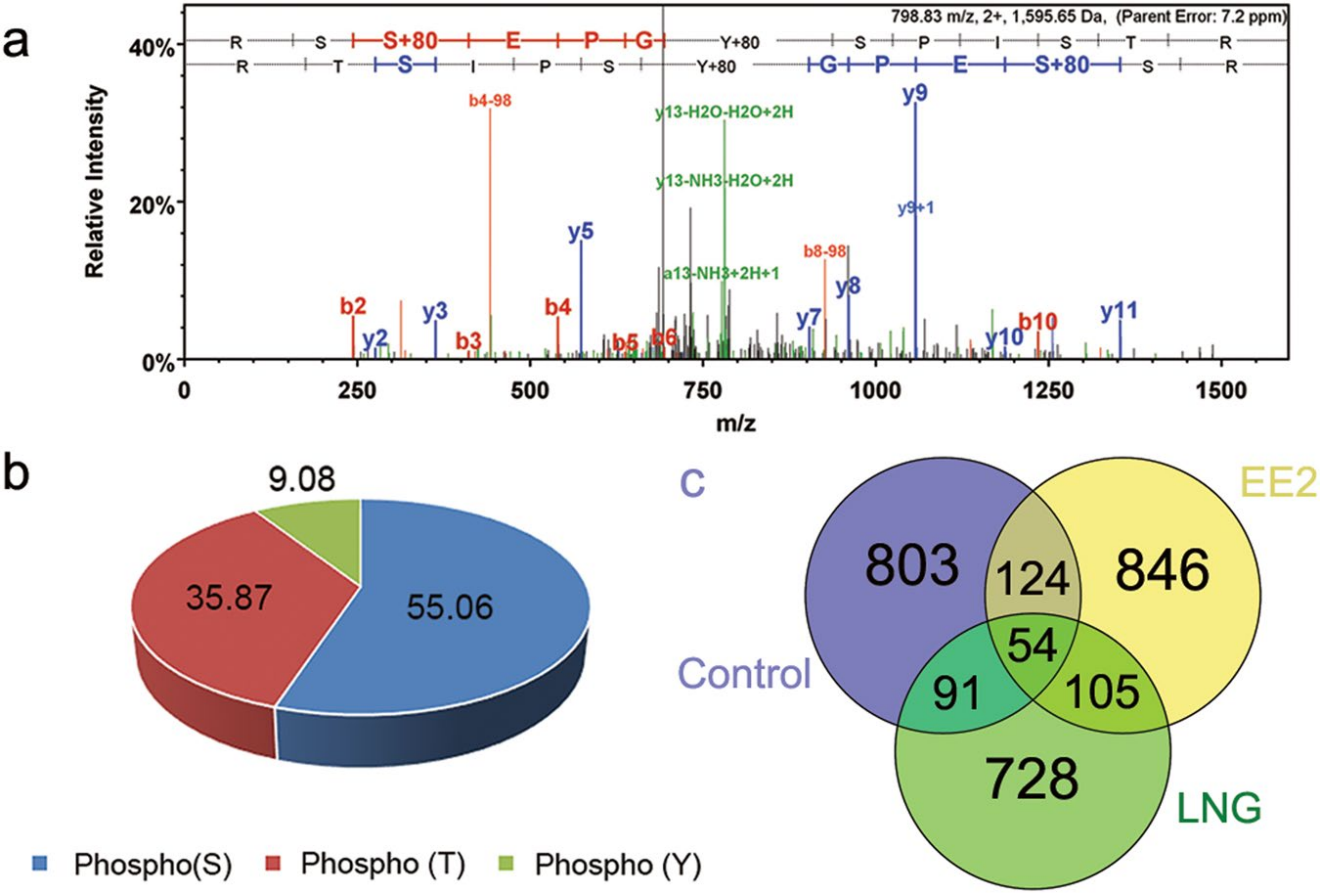
(**a**) Representative spectra of chondroitin sulfate proteoglycan 5B (CSPG5B). (**b**) Distribution of phosphorylated residues in control, EE2 and LNG treatments. (**c**) Distribution of phosphorylated proteins identified in the enriched fraction with 90% peptide confidence and 95% protein confidence.

In order to identify targets of rapid, non-genomic signaling pathways in FHM, we utilized a non-gel based, label-free mass spectrometric phosphoproteomic analysis of proteins isolated from the brains of male fish exposed to environmental relevant concentrations^19–21^ of EE2 and LNG, 5 ng/L or 100 ng/L, respectively, for 30 minutes (Fig. 1). To overcome the poor stoichiometry of phosphorylated peptides and enhance phospho-peptide identification by MS/MS, trypsinized peptides were enriched for phosphopeptides using TiO_2_ NuTip micro columns. Both the enriched fraction of peptides and the flow-through (unbound fraction) were analyzed by MS and resulted in an average of 15,588.8 ± 3,861.5 (Mean ± SD) spectra collected in the enriched fractions and 30,871.3 ± 868.8 (Mean ± SD) spectra collected in the flow-through fraction of each sample across all treatment groups (Fig. S1). Of the spectra collected in the enriched fractions, 72.68% ± 4.44%, 60.33% ± 21.35%, and 69.58% ± 0.95% were phosphorylated in the Control, EE2, and LNG groups, respectively, while 2.05% ± 0.47%, 2.18% ± 0.64%, and 1.65% ± 0.64% were phosphorylated in the flow-through fractions, respectively (Fig. 2a), indicating the efficiency of the NuTip microcolumns to capture phosphorylated peptides. Importantly, similar percentages of phosphorylated spectra were obtained for each treatment group (Fig. S2). A representative spectrum is depicted in Fig. 2a.

The distribution of phosphorylated residues among all treatment groups was 55.06% serines, 35.87% threonines, and 9.08% tyrosines (Fig. 2b). Overall, 1,119 phosphoproteins were identified in the Control group, 1,158 in the EE2-exposed group, and 937 in the LNG-exposed group. The distribution of all identified phosphoproteins by treatment group is depicted in the Venn diagram (Fig. 2c).

### Semiquantitative Analysis

**Figure 3:**
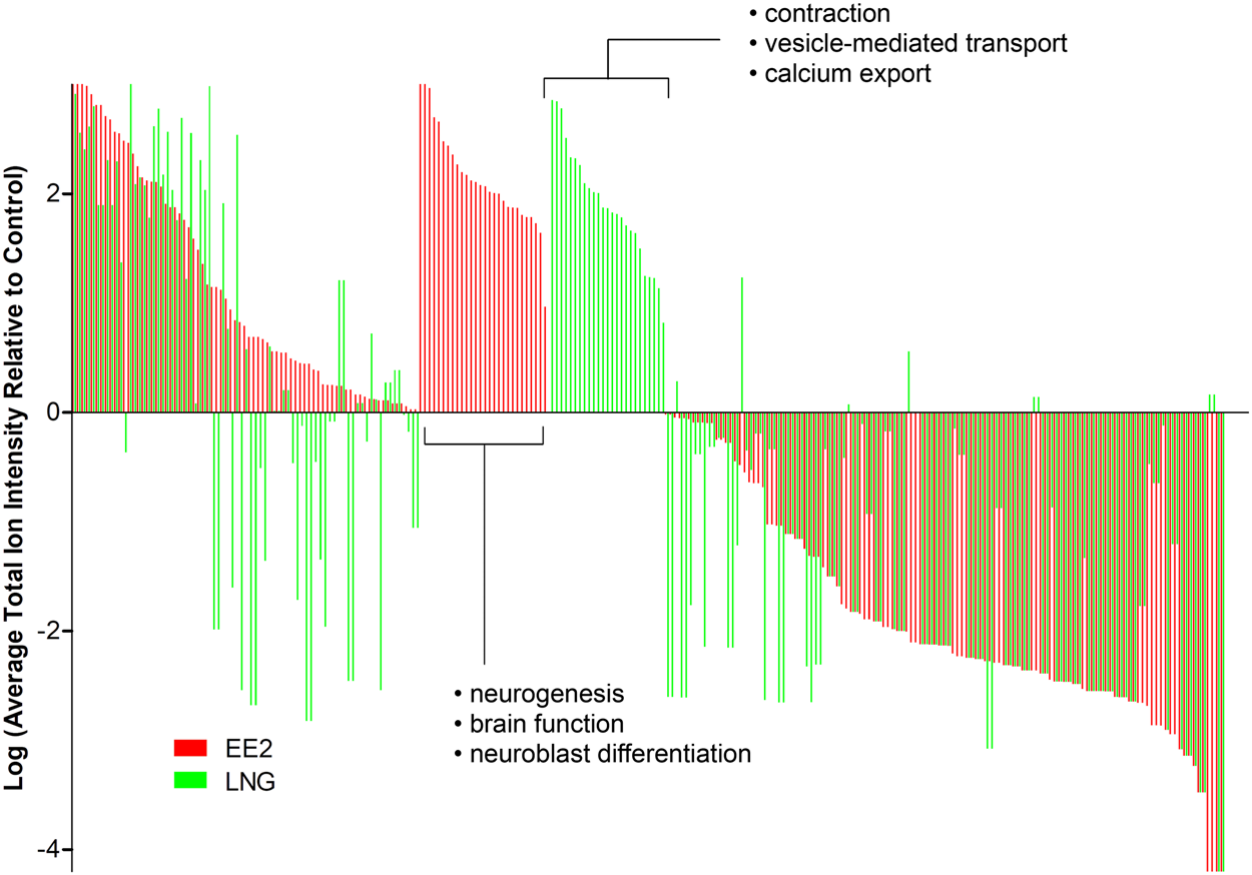
Log transformed average total ion intensity of confident identifications (CIs) by EE2 (red) and LNG (green) relative to control. Phosphoproteins were ordered from highest to lowest by EE2 with phosphoproteins specifically phosphorylated by each treatment in the middle. Text indicates cell processes that were significantly enriched (p < 0.05) with proteins between brackets as determined by subnetwork enrichment analysis using Pathway Studio 9 (Ariadne Genomics) operating on the ResNet 10.0 mammalian database.

A brief exposure of FHM to EE2 and LNG resulted in unique phosphorylation profiles in the brain. Phosphoproteins from enriched fractions were classified as confident identifications (CIs) if they were identified in at least two of the three replicates within at least one treatment group in a comparison. Altogether, there were 143 CIs in the EE2/Control comparison and 131 in the LNG/Control comparison yielding a total of 186 unique CIs among the two groups (Tables S2–S4). A semi-quantitative analysis of CIs based on the average total ion intensities of spectra matching a given phosphoprotein revealed a number of proteins that were differentially phosphorylated by EE2 and LNG relative to Control (Fig. 3). For clarity and consistency, we report the human homologs of the proteins identified in our analysis as these are required for downstream pathway analysis using Pathway Studio. Many phosphoproteins exhibited similar phosphorylation profiles in EE2 and LNG groups relative to controls including those that showed increased phosphorylation [(protein tyrosine phosphatase (PTPRJ), chondroitin sulfate proteoglycan 5 (CSPG5), enolase 1, alpha (ENO1)] and decreased phosphorylation [F-box and leucine-rich repeat protein 6 (FBXL6), podocalyxin-like (PODXL), growth factor receptor-bound protein 10 (GRB10), ubiquitin specific peptidase 37 (USP37), Snf2-related CREBBP activator protein (SRCAP), SIX homeobox 4 (SIX4), structural maintenance of chromosomes 6 (SMC6), trafficking protein particle complex 1 (TRAPPC1)]. Others exhibited increased phosphorylation specifically by EE2 [(nuclear cap binding subunit 3 (NCBP3), transient receptor potential cation channel, subfamily C, member 5 (TRPC5), Nance-Horan syndrome (NHS), otoferlin (OTOF), Mdm4 p53 binding protein homolog (MDM4), Establishment of cohesion 1 homolog 2 (ESCO2)] or LNG [myosin, heavy chain 6, cardiac muscle, alpha (MYH6), desmin (DES), voltage-dependent anion channel 3 (VDAC3), myeloid/lymphoid or mixed-lineage leukemia (MLLT3), G-protein coupled receptor 179 (GPR179)]. A number of phosphoproteins exhibited opposite directionality where EE2 increased phosphorylation and LNG decreased phosphorylation [myomesin 1 (MYOM1), SET domain, bifurcated 1 (SETDB1), dihydropyrimidinase-like 2 (DPYSL2), centrosomal protein 170 kDa (CEP170), sema domain, immunoglobulin domain (Ig), transmembrane domain (TM) and short cytoplasmic domain 4 C (SEMA4C)] or were increased by LNG but decreased by EE2 [capicua homolog (CIC), myosin, heavy chain 4, skeletal muscle (MYH4), storkhead box 2 (STOX2)] (Fig. 3 and Table S4). Of the proteins exhibiting increased phosphorylation only by EE2 relative to Control, many were sorted into cellular processes such as neuroblast differentiation, brain function, and neurogenesis; whereas LNG exposure resulted in increased phosphorylation of specific proteins involved in contraction, vesicle-mediated transport, and calcium export as determined by subnetwork enrichment analysis (p < 0.05) using Pathway Studio (Elsevier) (Fig. 3).

### Gene Ontology Analysis

**Figure 4:**
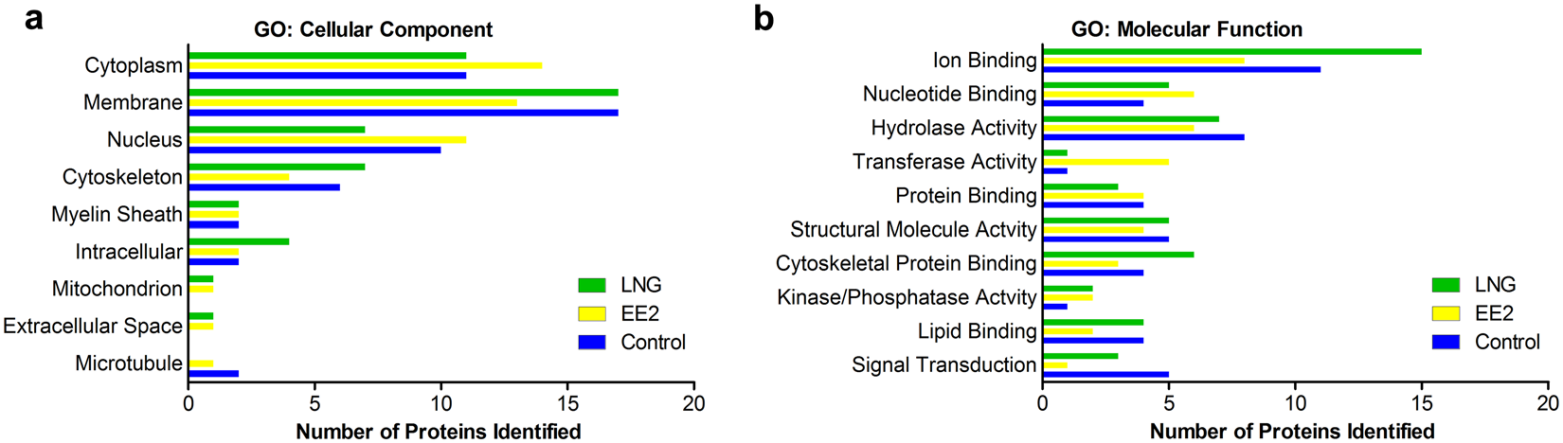
Gene Ontology analysis using GO Slim of the categories related to (**a**) cellular localization or (**b**) molecular function of CIs phosphorylated by CTL, EE2, and LNG.

In an effort to understand the broad types of phosphorylated proteins that were identified, we investigated gene ontology (GO Slim) categories for cellular localization and molecular function of the CI proteins from each group. Across all treatments, the nucleus, cytoplasm and membrane were the most represented cellular localizations (Fig. 4a). Exposure to EE2 phosphorylated more proteins located in the cytoplasm and fewer in the membrane when compared to Control and LNG. Alternatively, LNG exposure phosphorylated more proteins located in the cytoskeleton and fewer in the nucleus compared to Control and EE2. The molecular function of phosphorylated proteins was investigated as well; EE2 and LNG phosphorylated fewer proteins with general signal transduction activity compared to the Control group overall, however, both treatments resulted in phosphorylation of more proteins with kinase and phosphatase activity relative to Control suggesting an increase in phosphorylation mediated signaling as opposed to downstream receptor activation. Other molecular functions differed by treatment with EE2 exposure resulting in more proteins with transferase activity while LNG phosphorylated more proteins with cytoskeletal protein binding and ion binding activity (Fig. 4b).

### Subnetwork Enrichment Analysis

**Figure 5:**
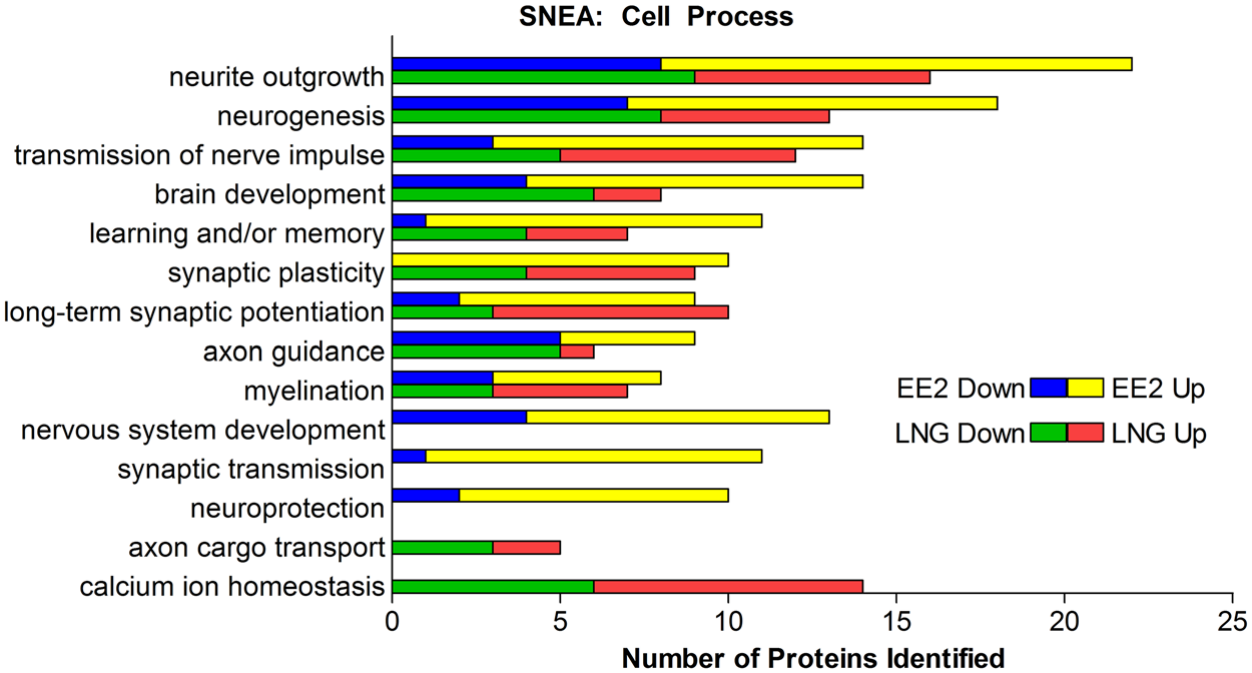
Neuro-related cell process subnetworks significantly over-represented by CIs (p ≤ 0.05). The number of proteins in each over-represented subnetwork with increased (Up) and decreased (Down) phosphorylations relative to control are indicated with red and green bars, respectively, in the EE2 group and yellow and blue, respectively, in the LNG group.

**Figure 6:**
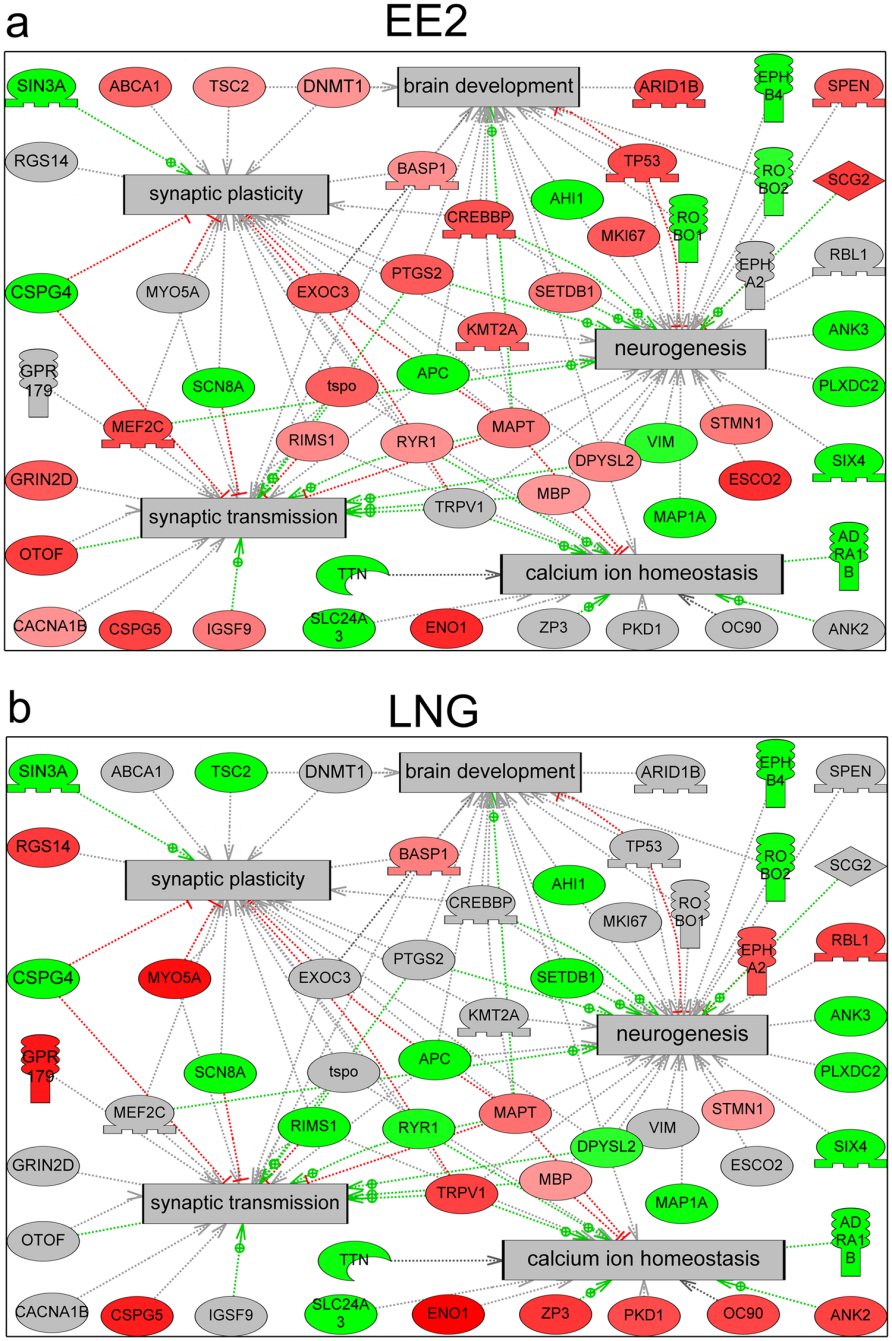
EE2 and LNG affected the phosphorylation of proteins involved in brain development, synaptic plasticity, neurogenesis, synaptic transmission, and calcium ion homeostasis although the specific phosphorylation profiles varied. Figures depict union pathways of proteins phosphorylated in response to EE2 (**a**) and LNG (**b**) and their interactions with significantly enriched (p < 0.05) cell processes (gray boxes) as determined by subnetwork enrichment analysis using Pathway Studio 9 (Ariadne Genomics) operating on the ResNet 10.0 mammalian database. Proteins exhibiting increased phosphorylation are colored red, proteins exhibiting decreased phosphorylation are colored green, and phosphoproteins that were not identified in a specific treatment are colored gray. Red linkages indicate up-regulation, green linkages indicate down-regulation, and gray linkages indicate unknown regulation.

In order to identify cell processes over-represented by CIs, a subnetwork enrichment analysis was performed on CIs relative to Control using Pathway Studio (Elsevier). In total, 140 and 102 cell process subnetworks were significantly enriched (p < 0.05) by differentially phosphorylated CIs for EE2 and LNG, respectively (Supplementary Table S4 and S5). Deeper analysis focused on cell processes related to the brain to identify rapid, non-genomic signaling targets of EE2 and LNG that would support the growing evidence of a role for sex hormone signaling in neuronal processes. EE2 exposure resulted in differential phosphorylation of 60 proteins or 42% of CIs that were sorted into subnetworks explicitly related to neuronal processes compared to 37 proteins or 30% of CIs in the LNG exposed group. Overall, EE2 exposure resulted in greater enrichment of cell processes relevant to the brain (22.1%) compared to LNG (18.6%) for significantly enriched subnetworks (p < 0.05). A number of neuronal cell processes were similarly represented by EE2 and LNG including synaptic plasticity, long-term synaptic potentiation, and myelination (Fig. 5). It’s important to note that while synaptic plasticity was similarly represented, all of the CIs identified in the EE2 group exhibited increased phosphorylation compared to Controls while LNG exhibited a mix of phosphoproteins with increased and decreased phosphorylations relative to Control (Fig. 5), although the precise consequence of increased or decreased phosphorylation on the function of these proteins is still unclear. EE2 exposure resulted in enrichment of unique cell processes such as nervous system development, synaptic transmission, and neuroprotection, while LNG affected the phosphorylation of proteins uniquely sorted into the cell processes of axon cargo transport and calcium ion homeostasis (Fig. 5). Of note, while both EE2 and LNG caused similar enrichment of cell processes such as synaptic plasticity, brain development, and neurogenesis, the specific suites of proteins that were differentially phosphorylated varied (Fig. 6a,b). In some cases, a protein was differentially phosphorylated by both EE2 and LNG but exhibited opposite directionality such as dihydropyrimidinase-like 2 (DPSYL2), regulating synaptic membrane exocytosis 2 (RIMS2), tuberous sclerosis 2 (TSC2), and ryanodine receptor 1 (RYR1) in synaptic plasticity, and SET domain, bifurcated 1 (SETDB1) in brain development (Fig. 6a,b).

## Discussion

The advent of’omics’ methodologies has made it feasible to characterize environmental chemicals into groups with known or novel mechanisms of action by surveying global changes in the transcriptome and proteome. In the future, these data can be used to more efficiently screen chemicals with the use of well described AOPs to compare against. However, it is growing more clear that in addition to studying changes in the transcriptome and the proteome to ‘reverse engineer’ AOPs, one must also consider changes in PTMs of proteins as these have been shown to highly and specifically regulate their function^22^. In particular, phosphorylation of proteins is emerging as a target for alteration by environmental contaminants^23,24^. The relevance of protein phosphorylation as a potential MIE in the AOP framework is highlighted by the proposal of a hypothetical AOP linking activation by phosphorylation of glycogen synthase kinase 3 beta (*gsk3b*) to impaired swim bladder inflation and population decline in fish^25^ and another identifying phosphorylation of acetyl choline esterase (AChE) as the MIE of AChE inhibition by organophosphate pesticides^24^. However, modulation of protein phosphorylation has only recently been considered as a potential key event in AOPs for HAAs, such as bisphenol-A (BPA), and has thus far only been presented in the context of human exposure^26^. Greater consideration is supported by the observations that several xenoestrogens are capable of activating membrane estrogen receptors with greater potency than 17β-estradiol (E2)^27^.

Given the minimal knowledge of specific phosphorylation events targeted by environmental contaminants, it is important to characterize global changes in phosphorylation to identify rapid signaling targets of chemicals in ecotoxicological studies, in efforts to move the field forward.

The development of non-gel based, label-free, mass spectrometric methods consisting of liquid chromatography coupled to tandem mass spectrometry (LC-MS/MS) for the analysis of protein phosphorylation has made it possible to identify many phosphorylated proteins simultaneously. This information can be used to construct plausible signaling networks based on interactions between differentially phosphorylated proteins thereby capturing a snapshot of non-genomic signaling mechanisms activated by a chemical exposure. This knowledge can be used to ‘reverse engineer’ an AOP to link a phenotypic response to potential key molecular events i.e. changes in phosphorylation^4^. The FHM represents the ideal species to link changes in phosphorylation to phenotypic events given the wealth of data regarding adverse effects in classical toxicological assays^28^. Using contemporary technology, we optimized a phosphoproteomic pipeline utilizing a phosphopeptide-specific enrichment method coupled to label-free LC-MS/MS. The analysis method was robust as evidenced by the low variability between MS/MS runs (Fig. S1), and our enrichment method was sensitive and specific as evidenced by the higher percentage of phosphopeptides in the enriched fractions compared to the flow-through fractions, both of which are common problems in label-free phosphoproteomic analyses^29,30^ (Fig. S2).

Limited studies have utilized LC-MS/MS based methods for the analysis of contaminant induced changes in phosphorylation in mammalian models and include an analysis of rat hepatoma cells exposed to 2,3,7,8-tetrachlorodibenzo-p-dioxin^12^, an analysis of oral administration of the environmental contaminant perfluorododecanoic acid in rat liver^13^, the effect of a mycotoxin on phosphorylation of proteins involved in innate immune responses in the mouse spleen^31^, and the modulation of arsenic toxicity to human kidney cells exposed to selenium^11^. To our knowledge, this is one of few studies to examine rapid changes in phosphorylation after such a brief exposure *in vivo* other than the study by Pan *et al*.^31^ and is certainly the first such study performed in fish. Further, no studies have probed the phosphoproteome as a target for environmental contaminants in ecotoxicological models using gel-free approaches as the only other phosphoproteomic analysis to our knowledge was performed in medaka fish (*Oryzias latipes*) exposed to mycrocystin-LR using 2D-gel electrophoresis coupled to LC-MS/MS^32^.

It is increasingly recognized that the brain of vertebrates, in particular teleost fish, is capable of neurogenesis in select regions, and that neurosteroids such as estradiol and progesterone are involved, reviewed by Pellegrini *et al*.^33^. Because the fish brain is a neuroendocrine active organ, it is a potential target for HAAs in the environment. Numerous synthetic compounds have been shown to influence neuronal signaling and cause changes in gene and protein expression in multiple developing and/or mature fish species including EE2^34–37^ and BPA analogs^38,39^, progesterone^40^, and the spironolactone derivative, drospirenone^41,42^. However, these studies focused on the classical, genomic mechanism of estrogen and progesterone action despite the measured expression of the membrane-bound G protein-coupled estrogen receptor (*gper1*)^43,44^ and membrane progesterone receptor (*pgr)*^45,46^ in the brains of various teleost species. Further, the ecological relevance of these studies is minimal as they primarily utilize zebrafish despite their limited environmental distribution and sparse data regarding adverse effects in classical toxicological assays^28^. The present study notably builds on this body of literature by investigating rapid signaling mechanisms of action of hormonally active chemicals in the more ecologically relevant species, the FHM.

Consistent with previous studies in mammals showing that estradiol and progesterone influenced neurogenesis and neuroprotection and modulated processes such as proliferation, migration, and apoptosis in the brain^47–53^, and others showing that EE2 influenced the expression of proteins involved in synaptic transmission in other teleost fish^34,37^, results of this work highlighted cell processes related to neurite outgrowth, neurogenesis, and synaptic plasticity, among others (Fig. 5), as targets of rapid signaling mechanisms activated by EE2 and LNG. Although both EE2 and LNG affected neuronal processes, the specific suites of proteins that were phosphorylated by each varied (Fig. 2), suggesting that they may have unique roles in the brain. Further, of those that were differentially phosphorylated compared to Control, many exhibited opposite directionality where EE2 increased phosphorylation while LNG exposure resulted in a decrease in phosphorylation, and vice versa (Figs 3 and 6). In regards to EE2, the enrichment of phosphoproteins involved in synaptic plasticity and synaptic transmission (Figs 5 and 6A) is intriguing because these processes are established targets of estrogen signaling in mammalian models^54–59^, but to our knowledge have not been identified as targets of rapid signaling pathways initiated by exposure to synthetic estrogens in the fish brain.

Our analysis also revealed interesting LNG-specific effects such as phosphorylation of proteins involved in calcium ion homeostasis (Figs 5 and 6B) which is particularly noteworthy as it has been shown that progesterone inhibition of neuronal calcium signaling underlies aspects of progesterone-mediated neuroprotection through the mitigation of inflammation, edema, demyelination, and excitotoxicity, reviewed by Luoma *et al*.^60^. However, LNG exposure did not result in a statistically significant enrichment of proteins involved in neuroprotection based on our pathway analysis, which may be due to the transient nature of phosphorylation or perhaps an effect on neuroprotection is not evident in such a brief exposure window (Fig. 5). Of note, LNG is derived from androgens^61^ and numerous studies now indicate that LNG may also act as an androgen receptor (*ar*) agonist at certain concentrations including the concentration used in this study. For example, LNG (≥40 ng/L) induced expression of spiggin, an AR target gene in three-spined sticklebacks (*Gasterosteus aculeatus*)^62^, at higher doses, LNG (100 ng/L) caused masculinization in FHMs demonstrated as de novo development of nuptial tubercles, a morphological characteristic of reproductively active males^63^, and elongation of anal fin rays, an androgen driven secondary sexual characteristic, in female eastern mosquitofish (*Gambusia holbrooki*)^64,65^. As such, the AOP linking androgen receptor activation to reproductive dysfunction in female FHMs (https://aopwiki.org/aops/23) is a plausible target for LNG as are membrane ARs expressed in the fish brain^66^. Future studies should investigate the precise contribution of membrane PGR and membrane AR in the observed phosphorylation events.

There exists extensive literature linking estrogen receptor agonism to reduction in cumulative fecundity and spawning, increased plasma vitellogenin concentrations, vitellogenin synthesis in liver, and renal pathology due to increased vitellogenin deposition^67^. This abundance of information is being used to develop an AOP linking estrogen receptor agonism to reproductive dysfunction in fish and is built on a number of studies utilizing 21 day toxicity tests and endpoints suspected to be modulated by the classical, genomic mechanism of estrogen action^67^. Similarly, most studies investigating a role for E2 and xenoestrogens in modulating brain function have focused on the estrogen induced up-regulation of the aromatase B gene (*cyp19a1b*)^68–70^ which has been shown to be mediated through activation of nuclear estrogen receptors and binding to estrogen response elements in the *cyp19a1b* promoter^71^. While we did not identify any transcription factors known for modulating *cyp19a1b* gene expression in the CI list, we identified closely related proteins including regulatory factor X3 (RFX3)^72^, CAMP responsive element binding protein 3 (CREB3)^73^, CAMP responsive element binding protein 5 (CREB5)^73^, and POU class 3 homeobox 2 (POUF3)^72^ in at least one sample. Results presented herein suggest that estrogen and progesterone receptor agonism, and potentially androgen receptor agonism, is capable of also activating rapid signaling mechanisms and may influence neurogenesis through phosphorylation of target proteins, potentially through activation of membrane-bound receptors. Future studies should be performed to confirm a requirement for membrane receptor activation in the observed changes in protein phosphorylation by utilizing membrane-impermeable ligands and membrane bound receptor specific inhibitors.

**Figure 7:**
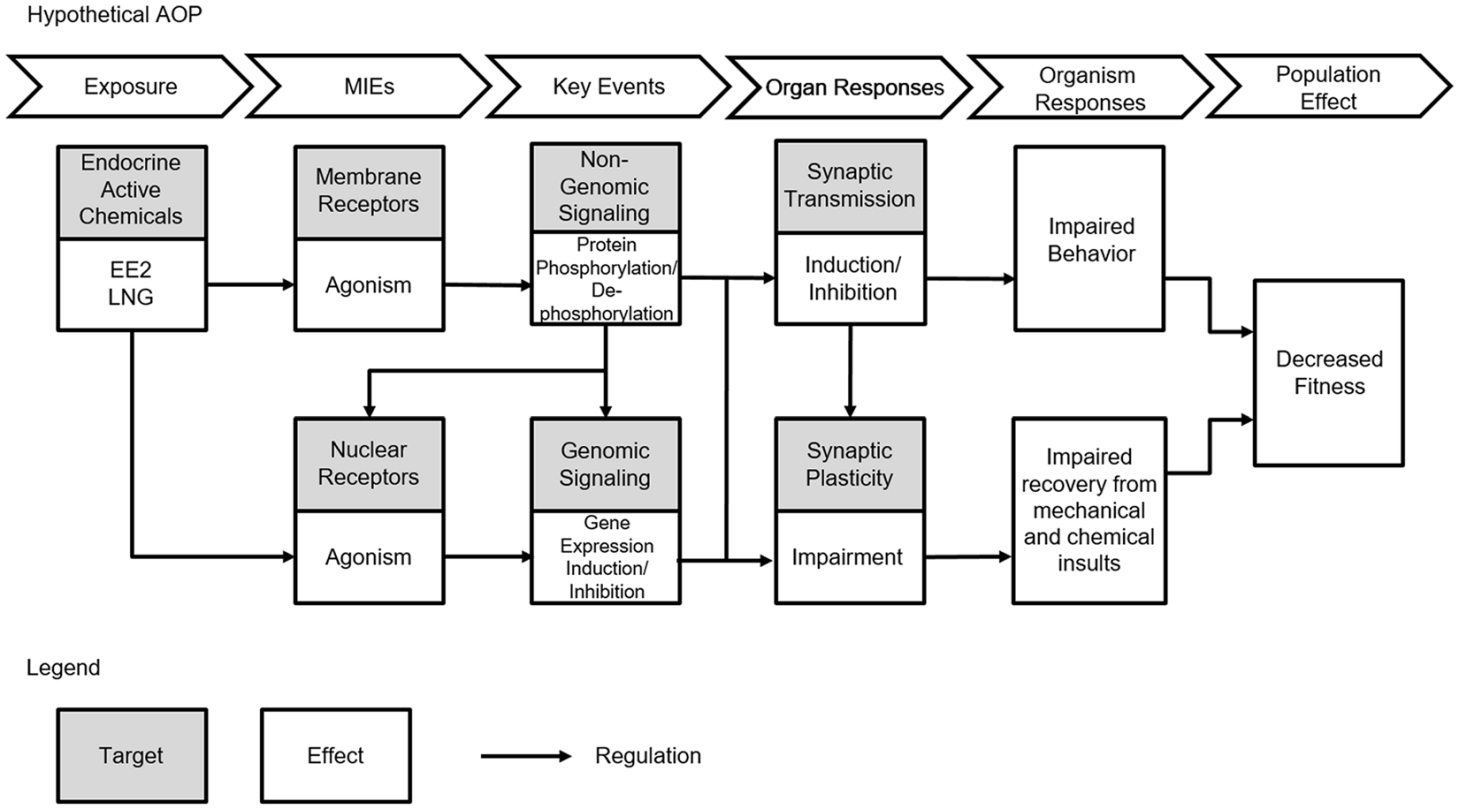
A hypothetical AOP depicting the interaction of both genomic and non-genomic receptor-mediated signaling pathways in response to contaminate exposure and how they may affect higher order responses.

Activation of progesterone receptors, either nuclear or membrane, by environmental progestins has yet to be incorporated into an AOP, and to our knowledge, activation of rapid signaling mechanisms and protein phosphorylation have not yet been considered as key events linking EE2 to adverse outcomes in any tissue. As such, we propose a hypothetical AOP constructed based on the published literature (Fig. 7) integrating activation of membrane receptors and nuclear receptors to direct future studies investigating adverse phenotypic responses influenced by rapid signaling mechanisms. In this AOP, exposure to HAAs present in the water causes activation and agonism of membrane and nuclear receptors^74,75^ which trigger activation of non-genomic and genomic signaling mechanisms that respectively modulate protein phosphorylation and gene expression. Activation of non-genomic signaling can also influence genomic signaling mechanisms^76^, particularly in the brain^77^, through modulation of nuclear receptor activity by direct phosphorylation of the receptor itself^78^ or phosphorylation of associated co-regulatory proteins^79^, and through activation of gene transcription at discrete promoters independent of nuclear receptors^79^. Differential phosphorylation of enzymes involved in synaptic transmission as is the case for organophosphate induced phosphorylation of AChE^24^ may lead to impaired behavior while induction of gene transcription such as *cyp19a1b* by nuclear receptors^71^ may influence the proliferative activity of the teleost brain and thus may impair the ability to recover from mechanical and chemical insults, all of which may result in decreased fitness. Certainly, these data set the foundation for future studies that directly link early rapid molecular changes from short chemical exposures to physiological and/or phenotypic endpoints.

Another underexplored area of ecotoxicological research is the site-specificity of protein phosphorylation which has been shown to affect hormone responses of the androgen receptor^80^ and to be discretely modulated by different agonists in immortalized cell lines^78^. This suggests that chemicals may exhibit a “phosphorylation fingerprint” that ultimately drives physiological and phenotypic changes. Unfortunately, databases containing site-specific phosphorylation information such as PhosphoSitePlus® are not yet compatible with our fish database due to divergent sequence information. Future mechanistic studies should probe the role of specific phosphorylation sites in the neuronal processes identified herein.

Future studies should also investigate mixture effects on protein phosphorylation profiles as the contaminants examined in this study often occur contemporaneously in aquatic environments, and there is evidence of additive effects of EE2 and LNG in brain aromatase bioactivity assays^81^ and evidence that estrogens up-regulate *pgr* expression in neurons and radial glial cells of fish^82^. To be sure, studies investigating multiple time-points, and phosphorylation profiles during critical developmental stages as receptor levels are known to fluctuate^44^, should also be pursued. As it is well known that hormonal effects in the brain are spatially distinct (reviewed by Pellegrini *et al*.^33^) future studies should probe differential phosphorylation of proteins in specific regions of the brain, Lastly, mechanistic studies connecting differential phosphorylation with apical endpoints are required to confirm a link between estrogen and progesterone receptor agonism, activation of rapid and potentially non-genomic signaling pathways, and phenotypic responses such as neurogenesis if they are to be used to develop adverse outcome pathways.

Overall, this work is the first to apply a non-gel-based LC-MS/MS approach to identify proteins that are differentially phosphorylated after a chemical exposure *in vivo* in fish, apply technology to a non-model species of overwhelming ecological relevance, and show that both EE2 and LNG are capable of modulating the phosphorylation of proteins in the brain of fish after a brief 30-minute exposure. While our study lacks an adverse phenotypic anchor, the data presented highlight the importance of considering activation of rapid signaling mechanisms mediated by phosphorylation events in addition to classical mechanisms of action of HAAs when constructing AOPs. Perhaps most importantly, this study may serve as a proof-of-concept of incorporating contemporary technologies to elucidate novel, tissue-specific mechanisms of action of HAAs in non-model species such as FHM.

## Materials and Methods

### Chemicals

17α-Ethinylestradiol (>98.0%, CAS 57-63-6) was purchased from Fluka and levonorgestrel (13-ethyl-17-ethynyl-17-hydroxy- 1,2,6,7,8,9,10,11,12,13,14,15,16,17- tetradecahydrocyclopenta[a] phenanthren-3-one; >98% purity, CAS 797-63-7) was purchased from Steraloids Inc. All chemicals were dissolved in a carrier solvent consisting of 0.005% triethylene glycol (TEG, Sigma) and 0.0003% ethanol (Acros) for the exposures.

### Exposures

All fish exposures were carried out with protocol approval by the UF Institutional Animal Care and Use Committee and all methods were carried out in accordance with relevant guidelines and regulations. Twenty-seven adult male fathead minnows (ca. 9 months old) were split into three treatment groups consisting of nine fish divided among three tanks (Fig. 1). Each treatment group was aqueously exposed to either 5 ng/L ethinylestradiol, 100 ng/L levonorgestrel, or carrier solvent control (three groups per treatment) for 30 minutes in 2 L beakers containing 600 mL milliQ water (pH 7.3–7.4) supplemented with 2.5 g synthetic sea salt (Instant Ocean SS15-10), and gentle aeration. Doses and time-points were chosen based on previous studies by our group and others^31,37,65,83,84^. After exposure, fish were euthanized by submersion in buffered 0.15 g/L tricaine-S (MS-222, Western Chemical) and whole brain tissue was harvested and flash frozen in liquid nitrogen thereafter. To reduce sampling bias, all exposures were performed on the same day and staggered to ensure uniform exposure and dissection timelines. Time from euthanasia to tissue freezing was consistently less than 5 minutes? Tissues from three fish were randomly pooled per treatment yielding three biological replicates per treatment to minimize the effects of experimental staggering.

### Protein Extraction

Pooled brain tissues were mechanically disrupted on ice in 200 μL RIPA extraction buffer (25 mM Tris-HCl pH 7.6, 150 mM NaCl, 1% nonyl phenoxylpolyethoxylethanol-40 (NP-40), 1% sodium deoxycholate and 0.1% SDS) (Pierce) containing a protease inhibitor tablet (proprietary formulation containing AEBSF HCl, aprotinin, bestatin, E-64, leupeptin, pepstatin, EDTA) (Pierce) and phosphatase inhibitor cocktail set I (2.5 mM bromotetramisole oxalate, 500 μM cantharidin, 500 nM microcystin-LR) and II (200 mM imidazole, 100 mM sodium fluoride, 115 mM sodium molybdate, 100 mM sodium orthovanadate, 400 mM tartrate dihydrate) (Calbiochem). Samples were clarified at 12,000 × g for 30 minutes at 15 °C and supernatants were transferred to 2 mL screw-cap tubes for precipitation. Samples were precipitated with 80% acetone/10% trichloroacetic acid (TFA) overnight at −20 °C. Precipitated proteins were pelleted by centrifugation at 20,000 × g for 30 minutes at 4 °C using an Eppendorf Centrifuge 5417 R, washed with 100% acetone, vortexed, and incubated on ice for 30 minutes. This was repeated with 80% acetone 2 × and then 80% ethanol. Washed, precipitated proteins were reconstituted in RIPA containing protease and phosphatase inhibitors. Total protein was determined by DC^TM^ protein assay kit II following the manufacturers protocol (Bio-Rad).

### Protein Digestion and Phosphopeptide Enrichment

Samples were precipitated with acetone and solubilized in 110 μL of ABC buffer (50 mM ammonium bicarbonate, pH 8.0). EZQ protein assay (Molecular Probes) was performed and 200 μg of protein aliquots were taken for trypsin digestion. Samples were digested with 1:50 (enzyme:protein) sequencing grade trypsin (Promega) and then a phosphopeptide enrichment was done with TiO_2_ NuTip micro columns (GlygenSci) following the method reported by Gates *et al*.^85^. Briefly, the microcolumns were equilibrated by washing the TiO_2_ resin with 5 μL binding solution (80% acetonitrile (ACN), 15% H_2_O, 5% TFA, pH < 3) and excess buffer was aspirated from the tip. The digested sample was mixed 1:1 with binding solution and loaded onto the tip by aspirating and expelling 2.5 μL aliquots ~ 20 times over several minutes. Next, the micro columns were washed with 80% ACN, 19% H_2_O, 1% TFA (pH < 3) and the bound peptides were eluted with 10 μL elution solution (2% NH_4_OH in water, pH = 11). Phosphopeptides were solubilized in 20 μL of loading buffer (3% ACN, 0.1% acetic acid, 0.01% TFA). An aliquot of 5 μL was taken and mixed with 5 μL of loading buffer. Flow-through samples were solubilized in 100 μL of loading buffer and prepared and a 5 μL aliquot was mixed with 5 μL of loading buffer.

### Mass Spectrometry

To ensure optimum coverage, phosphopeptides were analyzed on both a Q-Exactive plus mass spectrometer (Thermo Fisher Scientific) and a LTQ Orbitrap XL mass spectrometer (Thermo Fisher, Bremen, Germany). For analysis on Q-Exactive plus MS, a total of 10 μL of sample was injected to the nano LC-MS/MS using an automated Easy-nLC 1000 system coupled to a Q-Exactive plus MS. A pre-column (20 mm × 75 μm; 3 μm-C_18_) and an analytical column (500 mm × 75 μm; 2 μm-C_18_) were used (Thermo Fisher Scientific) with mobile phases A (0.1% formic acid in water) and B (0.1% formic acid in ACN). The phosphopeptides were separated at a flow rate of 300 nL/min using the following gradient: 2–25% mobile phase B from 0–95 min, 25–98% mobile phase B from 95–100 min, and 98% mobile phase B from 100–120 min. Data dependent mass spectra were acquired for 120 min for the top 5 peaks. The full MS surveys were collected over a mass-to-charge ratio (m/z) range of 400–2000 m/z, with the resolution set to 70,000, and Max IT 100 ms. For MS/MS, we used a resolution of 17,500 and Max IT 64 ms, with an isolation window of 2 m/z and NCE of 28.

For analysis on the LTQ Orbitrap XL MS, a total of 10 μL of sample was injected to a C_18_ capillary trap cartridge (LC Packings, United States), and separated on a 15 cm nanoflow analytical C_18_ PepMap column (0.075 mm inner diameter, 3 μm particle size, 100 Å) at a flow rate of 300 nL/min using a nanoLC ultra 1D plus system (AB Sciex, United States). For peptide separation solvent A was 3% (vol/vol) ACN and 0.1% (vol/vol) acetic acid. Solvent B was 97% (vol/vol) ACN and 0.1% (vol/vol) acetic acid. Peptide separation was performed using a linear gradient from 3 to 40% solvent B for 100 min, followed by an increase to 90% solvent B in 10 min and holding for 10 min. The flow was directly sprayed onto an LTQ Orbitrap XL MS. MS/MS spectra were acquired in a data-dependent mode. An Orbitrap MS full scan (resolution, 3 × 10^4^; molecular-mass range, 300 to 2000 Da) was followed by 10 MS/MS scans in the ion trap, which were performed by collision-induced dissociation on the top 10 most abundant ions. The isolation window for ion selection was 2 Da. The normalized collision energy was set at 35%. The dynamic exclusion time was 20 s. Additionally, if a phosphate neutral loss of 98, 49, 32.66, or 24.5 *m*/*z* below the precursor ion mass was detected, a multistage activation (MSA) event was repeated for the top five ions in a data-dependent manner provided the precursor exceeded a threshold of 6000 ion counts. The mass spectrometry proteomics data have been deposited to the ProteomeXchange Consortium via the PRIDE partner repository^86^ with the dataset identifier PXD007714.

### Database Searching and Protein Identification

A custom database was constructed for searching for protein identification. This database was a composite of an in-house FHM protein database and the zebrafish, *Danio rerio*, database on uniprot. The in-house FHM database was created by selecting the longest open reading frame from the 6-frame translation of each sequence in our transcriptome database in Blast2Go with the ORF predictor function. The software chose the longest open reading frame for each sequence, which was subsequently annotated against zebrafish NR database using blastx and blastp, resulting in 56,099 annotated sequences. Once combined with the uniprot zebrafish protein database our composite database consisted of 117,445 entities. Development of this custom composite database was essential to making high confidence protein identifications.

All MS/MS samples were analyzed using Mascot (Matrix Science, London, UK; version 2.4.1) and X! Tandem (The GPM, thegpm.org; version CYCLONE (2010.12.01.1)). Mascot was set up to search our custom composite database assuming the digestion enzyme trypsin. X! Tandem was set up to search a subset of the database also assuming trypsin. Mascot and X! Tandem were searched with a fragment ion mass tolerance of 0.50 Da and a parent ion tolerance of 10.0 PPM. Carbamidomethyl of cysteine was specified in Mascot and X! Tandem as a fixed modification. Gln->pyro-Glu of the n-terminus, deamidation of asparagine and glutamine, oxidation of methionine and phosphorylation of serine, threonine and tyrosine were specified in Mascot and!X-Tandem as variable modifications.

Scaffold (version Scaffold_4.4.1.1, Proteome Software Inc., Portland, OR) was used to validate MS/MS based peptide and protein identifications. Peptide identifications were accepted if they could be established at greater than 90.0% probability by the Scaffold local FDR algorithm. Protein identifications were accepted if they could be established at greater than 95.0% probability and contained at least 1 identified peptide which is widely accepted for phosphoproteomic studies which rely on fewer peptides for protein identification due to the substoichiometry of phosphorylated peptides relative to non-phosphorylated peptides^87,88^. Protein probabilities were assigned by the Protein Prophet algorithm^89^. Proteins that contained similar peptides and could not be differentiated based on MS/MS analysis alone were grouped to satisfy the principles of parsimony. Proteins sharing significant peptide evidence were grouped into clusters. Spectra were combined if they mapped to the same protein in different species in the database. Quantitation was performed based on average total ion current (AVG TIC). A phosphoprotein with AVG TIC ≤ 1 was considered to not be identified. In cases were a phosphoprotein was not identified in a particular group, 165.18 was imputed for calculation of a AVG TIC ratio as this was the smallest AVG TIC recorded similar to the method of Freund and Prenni^90^. Although it is recommended to normalize phosphoprotein abundance to total protein abundance to account for changes in expression^91^, we circumvented this by utilizing a short exposure window in which differential phosphorylation was assumed to be due to treatment specific changes in phosphorylation state and not due to changes in overall protein abundance as nuclear estrogen receptor-dependent translation is not expected to be induced after 30 minutes^92^. Phosphoproteins were mapped to human homologs (Table S1).

### Bioinformatics and Pathway Analysis

A list of confidently identified (CIs) proteins in each treatment group was created and included only phosphorylated proteins that were found in at least 2 of the 3 replicates. This list of CIs was used to conduct Gene Ontology (GO) analysis of cellular component and molecular function using GO Slim categories. Functional enrichment analysis was conducted in Pathway Studio 9 (Ariadne Genomics) operating on the ResNet 10.0 mammalian database using the Fisher’s Exact Test Subnetwork Enrichment Analysis option limiting subnetworks to those with p < 0.05.

### Significance Statement

A priority focus area of research in the field of ecotoxicology is linking molecular events initiated by contaminant exposure to higher-level effects on individuals and populations. The development of such frameworks, termed adverse outcome pathways (AOPs), have produced several well accepted schemes for hormonally active agents (HAA) which preferentially consider activation of nuclear receptor-mediated pathways as molecular initiating events (MIE). Inclusion of chemically induced rapid responses driven by non-genomic signaling by membrane receptors and mediated by protein phosphorylation has been minimally explored. These important mechanisms can represent an initial response of an organism to its environment and stimulate phenotypic changes. Results presented here establish activation of rapid signaling through phosphorylation events as MIEs that should be considered in the development of AOPs.

### Data Availability Statement

The authors declare no restrictions on the availability of materials or information.

## Electronic supplementary material

Supplementary Figures

Supplementary Tables
