# Supplementary material for: Early phosphoproteomic changes for adverse outcome pathway development in the fathead minnow (Pimephales promelas) brain
Source: Sci Rep. 2018 Jul 5;8:10212. doi: 10.1038/s41598-018-28395-w (PMC6033950; doi:10.1038/s41598-018-28395-w)
Supplement: Supplementary file 1 — Supplementary Figures [file 41598_2018_28395_MOESM1_ESM.docx]

*Title*: Early phosphoproteomic changes for adverse outcome pathway

development in the fathead minnow (*Pimephales promelas*) brain

Supplementary Data

*Author affiliation*: L.C. Smith^a,c*^, C.M. Lavelle^b,c*^, C. Silva-Sanchez^d^, N.D. Denslow^a,c^, and T. Sabo-Attwood^b,c^

a. Department of Physiological Sciences, University of Florida, 1333 Center Dr., Gainesville, FL

32603

b. Department of Environmental and Global Health, University of Florida, 1225 Center Dr., Rm

4160, Gainesville, FL 32610

c. Center for Environmental and Human Toxicology, University of Florida, 2187 Mowry Rd,

Gainesville, FL 32611

d. Interdisciplinary Center for Biotechnology Research, University of Florida, 2033 Mowry Rd,

Gainesville, FL 32601

*Co-first authors

Corresponding authors: Tara Sabo-Attwood, Department of Environmental and Global Health,

University of Florida, 1225 Center Drive, Box 100188, Gainesville, FL 32610, United States.

(352)-294-5293. [sabo@phhp.ufl.edu](mailto:sabo@phhp.ufl.edu)

Nancy Denslow, Department of Physiological Sciences, University of Florida, 1333 Center Dr.,

Gainesville, FL 32603, United States.

(352) 294-4642. ndenslow@ufl.edu

**Figure S1**

**

**

Supplementary Figure 1. Total amount of spectra collected. Spectra collected in enriched and flow-through fractions from LTQ-Orbitrap XL and Q-Exactive Plus were combined. Bars are Mean ± SD.

**Figure S2**

**

**

Supplementary Figure 2. Enrichment of phosphorylated spectra. Percent of the proteins identified with 90% peptide confidence and 95% protein confidence that were phosphorylated in the enriched and flow through fractions. Bars are Mean ± SD. Enriched and Flow-though were compared for differences using a t-test, ** denotes p<0.01 and *** denotes p<0.0001
